# Supplementary material for: Plasma exosomes in insulin resistant obesity exacerbate progression of triple negative breast cancer
Source: BMC Cancer. 2025 Jul 9;25:1089. doi: 10.1186/s12885-025-14447-8 (PMC12239313; doi:10.1186/s12885-025-14447-8)
Supplement: Supplementary file 4 — Supplementary Material 4. [file 12885_2025_14447_MOESM4_ESM.docx]

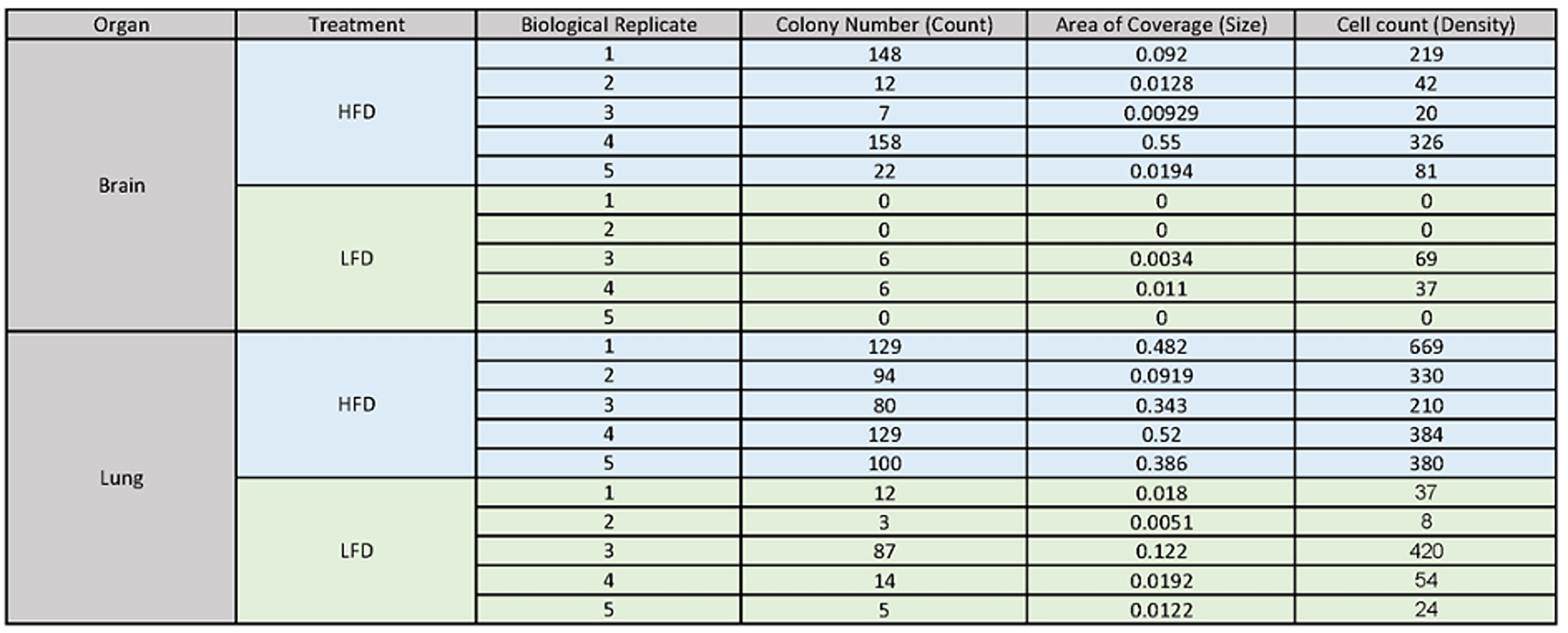


**Supplementary Table T3: Effects of HFD and LFD on Colony Formation in Brain and Lung Organs.**

Table presents data from biological replicates of brain and lung tissues obtained from C57BL/6J mice injected with E0771-GFP cells pre-treated for 72 hours with either HFD or LFD-plasma derived exosomes. Colony formation, quantified by colony number, was significantly higher in HFD-treated brain and lung samples compared to their LFD counterparts. Specifically, the brain samples from HFD-treated subjects showed large colony counts, with a peak in replicate 4 (158 colonies), while LFD-treated brain replicates either exhibited no detectable colonies (replicates 1 and 2) or very low counts (6 colonies in replicates 3 and 4). Similarly, in lung tissues, replicates treated with an HFD consistently had higher colony numbers, with replicate 1 showing the maximum count (129 colonies), compared to much lower counts in LFD-treated lung replicates, with the highest colony count being only 87 (LFD biological replicate number 3).
